# Religiosity, Religious Coping and Distress Among Outpatients with Psychosis in Singapore

**DOI:** 10.1007/s10943-022-01596-4

**Published:** 2022-06-25

**Authors:** Laxman Cetty, Anitha Jeyagurunathan, Kumarasan Roystonn, Fiona Devi, Edimansyah Abdin, Charmaine Tang, Swapna Verma, Siow Ann Chong, Jonathan Ramsay, Mythily Subramaniam

**Affiliations:** 1 Research Division, Institute of Mental Health, Buangkok Green Medical Park, 10 Buangkok View, Singapore, 539747 Singapore; 2 Department of Psychosis, Institute of Mental Health, Buangkok Green Medical Park, 10 Buangkok View, Singapore, 539747 Singapore; 3 College of Healthcare Sciences, James Cook University, 149 Sims Drive, Singapore, 387380 Singapore; 4 Lee Kong Chian School of Medicine, Nanyang Technological University, 11 Mandalay Road, Singapore, 308232 Singapore

**Keywords:** Religious coping, Religiosity, Spirituality, Psychosis, Singapore

## Abstract

This study aimed to investigate the prevalence of religious coping and explore the association between religious coping, religiosity, and distress symptoms amongst 364 outpatients diagnosed with psychosis in Singapore. Positive and Negative Religious Coping (PRC and NRC), religiosity (measuring the constructs of Organised Religious Activity (ORA), Non-Organised Religious Activity (NORA), and Intrinsic Religiosity (IR)) and severity of distress symptoms (depression, anxiety and stress) were self-reported by the participants. The majority of participants (68.9%) reported religion to be important in coping with their illness. Additionally, multiple linear regression analyses found that NRC was significantly associated with higher symptoms of distress. In contrast, ORA was significantly associated with lower anxiety symptom scores. Overall, the study indicates the importance of religion in coping with psychosis and the potential value in incorporating religious interventions in mental health care.

## Introduction

Religion, defined as an organised and institutional structure involving rituals, doctrines, and dogmas related to the sacred or the divine (Russinova & Cash, 2007), has been reported to play an important role in the lives of patients with mental illnesses. However, while on the one hand, positive associations between religiosity and well-being (Koenig, 2012) and physical health (Seybold & Hill, 2001) have been documented, religion has also been shown to worsen well-being and health (Pargament & Lomax, 2013).

Given the centrality of religion in the lives of many adherents, it is unsurprising that religious coping was found to be highly prevalent among individuals who have psychosis. For example, in a sample of consecutively admitted inpatients with psychosis, Kirov and colleagues (Kirov et al., 1998) observed that 61% adopted religious coping when dealing with their disorder. Additionally, patients with schizophrenia and psychosis have tended to attribute religious elements to be the cause of their illness (Saravanan et al., 2007) and play an important part in their recovery journey (Heffernan et al., 2016).

Religious coping refers to “the use of religious beliefs or behaviours to facilitate problem-solving to prevent or alleviate the negative emotional consequences of stressful life circumstances” (Koenig et al., 1998). One of the best-established findings in the extant literature is the distinction between positive and negative religious coping, often abbreviated to PRC and NRC, respectively (Pargament et al., 1998). Positive religious coping is characterised by “religious forgiveness, seeking spiritual support, collaborating religious coping, spiritual connection, religious purification, and benevolent religious appraisal”, while NRC is defined by “spiritual discontent, punishing God reappraisals, interpersonal religious discontent, demonic reappraisal, and reappraisal of God’s powers” (Pargament et al., 1998). Studies have shown that PRC can be protective and aid in one’s recovery from schizophrenia (Webb et al., 2011) and predicts better quality of life, social functioning, and clinical global impression (Mohr et al., 2011). It is also associated with greater reductions in depression and anxiety symptom scores (Rosmarin et al., 2013). Conversely, NRC has been shown in studies to contribute to a lower quality of life (Nolan et al., 2012), high levels of depression and anxiety (Nurasikin et al., 2013; Rosmarin et al., 2013), and conflict with psychiatric treatment (Mohr et al., 2006).

While religious coping through PRC and NRC includes indices that measure how one might use religion to cope cognitively through thoughts and attitudes, another way to investigate the relationship between religion and distress symptoms might be through understanding one’s religiosity. The five-item measure of religiosity: the Duke University Religion Index (DUREL), identifies three dimensions of religiosity: organisational religious activity (ORA), non-organisational religious activity (NORA), and intrinsic religiosity (IR; Koenig et al., 1997). ORA assesses public religious activity such as attending public religious services, while NORA assesses private religious activity such as prayer or reading religious scriptures. IR, on the other hand, examines the degree of personal religiosity or motivation. An intrinsically religious person identifies religion as their master motive, and all other dealings in life are brought into harmony with these beliefs (Allport & Ross, 1967). Thus, IR involves the pursuit of religion as an ultimate end in itself, as opposed to other extrinsic forms where religion is used as a means to something else (e.g., financial rewards or social status) (Koenig & Büssing, 2010). Currently, there seems to be a lack of research on the impact of religiosity on distress symptoms in populations with psychosis. However, in one of the few studies to date, Nurasikin and colleagues (2012) found that all three religiosity measures predicted lower overall distress symptom scores among psychiatric outpatients, including those with psychosis. In all, understanding one’s religious beliefs and attitudes and how individuals are committed to their religious beliefs and attitudes might be important in creating a complete religious profile and possibly inform clinicians in planning or referring clients for religious interventions. Religious interventions (RIs) include involvement of religion in psychotherapy, meditation, and pastoral services. A recent systematic review and meta-analysis of randomised controlled clinical trials investigating RIs in mental health care indicated an association of RIs with reductions in clinical symptoms such as depression and anxiety (Gonçalves et al., 2015).

Most of the extant research investigating the role of religion in patients with psychosis has been conducted in Western populations (Mohr et al., 2011; Nolan et al., 2012; Rosmarin et al., 2013). The present research sought to address this gap by examining the role religion plays among those with psychosis in a multi-ethnic Asian country, Singapore. Singapore is a city-state-country in South East Asia, with a population of about 4.02 million citizens and permanent residents, with the majority comprising those of Chinese (74.3%), followed by Malay (13.5%) and Indian ethnicities (9.0%) (Department of Statistics, 2021). Notably, the 2020 Census Report by the Department of Statistics (2021) highlighted the religious diversity in the population, with the majority having Buddhist (31.1%), no religion (20.0%), Christian (18.9%), and Islamic (15.6%) affiliations. Among those of Chinese ethnicity, Buddhism (40.4%) was the dominant affiliation, followed by no religion (25.7%) and Christianity (21.6%). Among the Malay demographic, however, Islam was almost ubiquitously represented (98.8%). In the Indian demographic, there was some diversity, with the majority having Hindu (57.3), Islam (23.4%), and Christian (12.6%) affiliations. Among Buddhism, the Theravada, Chinese Mahayana, and Tibetan Vajrayana were the prominent forms in Singapore (Chia, 2009). For the Christians, the majority were Protestants, Orthodox, and those who classified themselves as “Other Christianity” in the survey (Mathews et al., 2019). The majority of Muslims in Singapore were Sunni Muslims who subscribe to the Shafi’i’ school of thought (Bin Abbas, 2012). Given the existence of several religious belief systems and communities, it is not a surprise that religious syncretism, conceptualised as “hybridisation” and “transfiguration,” has been proposed to exist among the “Chinese religions” consisting of Taoism, Buddhism, and Confucianism (Goh, 2009). All in all, Singapore presents an opportunity for investigation in a unique mix of ethnic groups and religious diversity that has been missing from research that has focused largely on western and protestant majority populations.

Specifically, the current research aimed to: ascertain the prevalence of using religion in coping with psychosis and, investigate the relationship between religious coping patterns, religiosity, and distress symptoms. While this research is exploratory, it was hypothesised that (1) the majority of patients would report religion to be important in coping with their illness and that (2) positive religious coping would be associated with less distress while negative religious coping would be associated with greater distress.

## Methods

### Sample

The present research was conducted with outpatients who were seeking treatment at a tertiary psychiatric hospital, the Institute of Mental Health (IMH), in Singapore. The inclusionary criteria were that participants must be (a) Singapore citizens or permanent residents, (b) literate in English, (c) aged between 21 and 65 years, (d) diagnosed with any DSM-5 (American Psychiatric Association (2013) psychotic disorder or mood disorder with psychotic features except substance-induced psychotic disorder, and (e) not diagnosed with any intellectual disability. Participants were recruited over a period of 15 months from January 2018 to April 2019.

Recruitment of participants was done using a combination of two methods: (1) attending clinicians referred interested patients to the study team, (2) study flyers were posted in the waiting areas of the clinic with relevant information about the study eligibility criteria and procedures along with the contact details of the study team members. Trained study team members, who were not directly involved in the treatment of the participants, explained the study procedures and obtained written informed consent from the participants. Participants then completed the self-report study questionnaire using a pencil and paper format. The data were entered into and subsequently managed using the REDCap^1^ electronic data capture tools hosted at the National Healthcare Group servers (NHG; Harris et al., 2009, 2019). The study questionnaire included participants’ socio-demographic information such as age, gender, and ethnicity, followed by the measures reported in the subsequent sections. Ethical approval for the study was obtained from the NHG Domain Specific Review Board (DSRB) and the IMH Institutional Research Review Committee (IRRC). All participants were provided an inconvenience fee of $40 SGD at the end of their participation. Participants, on average, took 40 minutes to complete the required study procedures.

### Measures

#### Religious Beliefs and Religiosity

Data on participants’ religious affiliation were obtained from the self-reported questionnaire. Participants were asked: “What is your religious preference?”. Possible response options were: “Baha’ism”, “Buddhism”, “Catholicism”, “Christianity”, “Hinduism”, “Islam”. “Jainism”, “Judaism, “Sikhism”, “Taoism”, “Zoroastrianism”, “Atheism”, “Agnosticism”, and “Others” with an open text to specify. For the purpose of this study, participants who reported “Atheism”, “Agnosticism” or “Free-thinker” in the open text for the “others” option to the question on their religious affiliation, were re-categorised as “No affiliation” group. Those who reported “Catholicism” (*n* = 16) were subsumed under “Christianity”. Additionally, those who reported “Taoism” (*n* = 12) were merged with those who reported “Buddhism” under the category of “Buddhism/Taoism” given that many in Singapore who worship Taoist deities and/or follow Taoist practices also worship Buddha and/or follow Buddhist teachings (Sinha, 2008).

A question on belief in God / a Higher Power adapted from Rosmarin and colleagues (2013) was asked; ‘To what extent do you believe in God / a Higher Power?’. A 5-point Likert-type scale response was used, ranging from ‘Not at all’ to ‘Very much’. Two questions adapted from Huguelet and colleagues (2006) were also asked; ‘How important is religion in coping with your illness?’ (“of no importance”, “of some importance”, “important”, “very important” or “essential”) and ‘Do you think religion is incompatible with psychiatric treatment?’ (“Yes” or “No”). For the purpose of the current study, those who reported “of no importance” or “of some importance” to the question on the importance of religion in coping with illness were categorised under the sub-category of “religion is not important in coping with illness” and those who reported otherwise were categorised under the sub-category of “religion is important in coping with illness.” These two sub-categories were levels assigned to the variable: “attitude towards religious coping.” Participants who answered “Not at all” to the question “To what extent do you believe in God / a Higher Power?” and “Of no importance” to the question “How important is religion in coping with your illness?” were directed to skip the sections on religiosity and religious coping.

The Duke University Religion Index (DUREL; Koenig et al., 1997) is a 5-item, multidimensional measure of religiosity. The five items assess three important forms of religiosity: Organisational Religious Activity (ORA; one item), Non-Organisational Religious Activity (NORA; one item); and Intrinsic Religiosity (IR; three items). Possible scores on both the ORA and NORA subscales range from 1 to 6, while possible scores on the IR subscale score range from 3 to 15. As suggested for non-western samples by Koenig and colleagues (1997), the current study made amendments to the original scale. Additional examples of “temple” and “mosque” were added to the first item of the DUREL scale. The term “Bible study” was replaced with “reading religious scriptures,” and examples of “Bible,” “Koran,” “Bhagavad Gita,” and “writings of Buddha” were also added to the second item. The DUREL has shown good test–retest reliability, internal consistency, and validity (Koenig & Büssing, 2010). The overall scale (Cronbach’s alpha = 0.81) and the IR subscale (Cronbach’s alpha = 0.83) indicated high internal consistency in the current study.

#### Religious Coping

The Brief RCOPE is a 14-item measure of the extent to which positive and negative religious coping methods are used to cope with major life stressors (Pargament et al., 1998). The measure comprises seven items that assess PRC and seven items that assess NRC. Each item is measured on a four-point scale anchored at 0 (“not at all”) and 3 (“a great deal”). The resulting scores for PRC and NRC consequently range from 0 to 21. Similar to previous studies using non-western samples (Gardner & Cabral, 1990; Khan & Watson, 2006), the term “church” was replaced with “religious community” to avoid the risk of bias to certain religions. The term “God” was replaced with “God / a Higher Power” to address similar concerns over appropriateness for polytheistic and nontheistic religions. The internal consistency was found to be high in the current study for both PRC and NRC (Cronbach’s alpha for PRC = 0.94, NRC = 0.86).

#### Distress

The 21-item version of the Depression Anxiety Stress Scale (DASS-21; Antony et al., 1998) measures participants’ severity of negative emotional states of depression, anxiety, and stress. It contains three subscales of depression, anxiety, and stress, with each measure consisting of 7 items. Participant responses were scored on a four-point Likert scale ranging from “never” to “almost always.” Each of the subscale scores ranges from 0 to 21. All subscales showed high consistency in the current study (Cronbach’s alpha for depression = 0.90, anxiety = 0.83, stress = 0.90).

### Clinical Information

The research team members extracted clinical information such as psychiatric diagnosis and age of onset of psychosis by reviewing patients’ medical records. For the purpose of this study, the patient’s primary diagnosis was categorised as schizophrenia and related psychosis or affective psychosis. The duration of participants’ illness was calculated by subtracting the age of onset of psychosis from their age when they participated in the study.

### Statistical Analyses

The socio-demographic and clinical information of the study sample, and the prevalence of religious coping and religiosity, were reported using descriptive statistics. Mean, standard deviations and range of scores were calculated for continuous variables and frequencies and percentages for categorical variables. As this was an exploratory study, correlation analyses (Pearson’s product momentum for correlation between continuous variables, point-biserial for correlation between binary and continuous variables and phi coefficient for correlations between binary variables) were conducted to determine any significant correlations with the dependent variables for the later regression analyses. Lastly, three linear regression analyses were conducted to investigate the relationship between the independent variables of religiosity (ORA, NORA, IR), religious coping (PRC, NRC), and the three dependent variables of depression, anxiety, and stress symptoms, after adjusting for significant socio-demographic and clinical variables from the previous correlation analyses. Statistically significant differences were evaluated at *p* < 0.05, using two-sided tests for the correlation analyses. For the three regression analyses, significant differences for *p* values were corrected to < 0.017 (or 0.05/3). All descriptive statistical analyses and correlation analyses were conducted using SPSS Statistics version 25 (IBM Corp., 2017). The multiple linear regressions were conducted with R Studio (R Core Team, 2019).

## Results

After screening, a total of 374 participants out of the 671 eligible participants approached (55.7% response rate) completed the study, of which 10 participants were excluded due to incomplete data. The remaining 364 participants included in the study had a mean age of 35.2 years (SD = 10.8), with the majority being of Chinese ethnicity (69.2%). Over half the sample belonged to the 21–34 years age group (54.1%), with most reporting themselves to be single (74.7%), and with pre-tertiary education (46.7%). The sample was distributed approximately equally in terms of gender. The most frequently reported religious affiliations in the current sample were: Christianity (29.7%), Buddhism/Taoism (25.8%), Islam (20.1%), and no affiliation (14.0%). The socio-demographic and clinical profiles of the participants are reported in Table 1.

**Table 1:** Socio-demographic and clinical information of participants

Characteristic	Total (*n* = 364)		Religion is important in coping with illness (*n* = 250, *%* = 68.7)		Religion is not important in coping with illness (*n* = 113, *%* = 31.0)	
	*n* (*%*) / range	Mean (*SD*)	*n* (*%*) / range	Mean (*SD*)	*n* (*%*) / range	Mean (*SD*)
*Gender*						
Male	168 (46.2)		111 (66.1)		57 (33.9)	
Female	196 (53.8)		139 (70.9)		56 (28.6)	
*Age group (years)*	21–65	35.2 (10.8)	21–65	36.3 (10.9)	21–64	32.8 (10.3)
21- 34	197 (54.1)		129 (65.5)		67 (34.0)	
35 – 44	93 (25.5)		61 (65.6)		32 (34.4)	
45 – 54	42 (11.5)		35 (83.3)		7 (16.7)	
55 – 65	29 (8.0)		24 (82.8)		5 (17.2)	
*Ethnicity*						
Chinese	252 (69.2)		157 (62.3)		95 (37.7)	
Malay	57 (15.7)		51 (89.4)		6 (10.5)	
Indian	40 (11.0)		29 (72.5)		11 (27.5)	
Others	15 (4.1)		13 (86.7)		1 (6.7)	
*Relationship status*						
Single	272 (74.7)		183 (67.3)		89 (32.7)	
Married / In a relationship	71 (19.5)		51 (72.9)		19 (26.8)	
Divorced / Separated / Widowed	21 (5.8)		16 (76.2)		5 (23.8)	
*Highest education level*						
Primary and below	9 (2.5)		6 (66.7)		3 (33.3)	
Secondary	93 (25.5)		70 (75.3)		22 (23.7)	
Pre-tertiary	170 (46.7)		118 (69.4)		52 (30.6)	
Tertiary	92 (25.3)		56 (60.9)		36 (39.1)	
*Religious affiliation*						
Christianity	124 (34.1)		104 (83.9)		20 (16.1)	
Buddhism / Taoism	94 (25.8)		56 (59.6)		38 (40.4)	
Islam	73 (20.1)		65 (89.0)		7 (9.6)	
Hinduism	15 (4.1)		13 (86.7)		2 (13.3)	
Sikhism	2 (0.5)		1 (50.0)		1 (50.0)	
No affiliation	51 (14.0)		7 (13.7)		44 (86.3)	
*Primary diagnosis*						
Schizophrenia and related psychoses	340 (93.7)		232 (68.2)		107 (31.5)	
Affective psychosis	23 (6.3)		17 (73.9)		6 (26.1)	
Duration of illness (years)	0–41	8.4 (8.9)	0–41	5.0 (9.3)	0–38	6.2 (7.5)
*Extent of belief in God / Higher Power*						
Not at all	22 (6.0)		1 (4.5)		21 (95.5)	
Very little	26 (7.1)		7 (26.9)		19 (73.1)	
Undecided	30 (8.2)		4 (13.3)		26 (86.7)	
Somewhat	96 (26.4)		62 (64.6)		34 (35.4)	
Very much	190 (52.2)		176 (92.6)		13 (6.8)	
*Importance of religion in coping with illness*						
Of no importance	56 (15.4)					
Of some importance	57 (15.7)					
Important	78 (21.4)					
Very important	75 (20.6)					
Essential	97 (26.6)					
*Belief that religion is incompatible with psychiatric treatment*						
No	252 (69.2)		176 (69.8)		76 (35.2)	
Yes	107 (29.4)		71 (66.3)		36 (33.6)	
*Duke University Religion Index (DUREL)*						
Organised Religious Activity (ORA) score	1–6	3.5 (1.4)	1–6	3.8 (1.4)	1–6	2.8 (1.4)
Non-Organised Religious Activity (NORA) Score	1–6	4.1 (1.8)	1–6	3.3 (1.7)	1–6	1.7 (1.2)
Intrinsic Religiosity (IR) Score	3–15	7.6 (3.4)	3–15	11.5 (2.7)	3–15	7.5 (3.3)
*Patterns of religious coping*						
Positive Religious *Coping (PRC)*	7–28	18.5 (6.9)	7–28	20.7 (5.9)	7–28	12.7 (5.7)
Negative Religious Coping (NRC)	7–28	12.3 (5.3)	7–28	12.7 (5.4)	7–25	11.2 (4.9)
*Depression, Anxiety and Stress Scale – 21 (DASS21) Score*						
Depression symptoms	0–42	11.2 (10.3)	0–42	10.5 (9.8)	0–42	12.7 (11.1)
Anxiety symptoms	0–40	10.3 (8.9)	0–40	10.3 (9.1)	0–36	10.1 (8.3)
Stress symptoms	0–42	11.5 (9.3)	0–42	11.4 (10.0)	0–36	11.5 (10.1)

Percentages reported in the “religion is important in coping with illness” and “religion is not important in coping with illness” columns are reported row-wise

3 cases of missing information for age group

5 cases of missing information for religious affiliation

1 case of missing information for the question on importance of religion in coping with illness

5 cases of missing information for the question on belief that religion is incompatible with psychiatric treatment

### Extent of Belief in God or a Higher Power, the Importance of Religion in Coping with Illness, and Prevalence of Religiosity

In the sample, 78.6% (*n *= 286/364) of participants answered at least “somewhat” to the question if they believe in a God or a Higher Power (6.0%: Not at all, 7.1%: Very little, 8.2%: undecided, 26.4%: somewhat, 52.2%: very much). In addition, 68.9% (*n* = 250/363) of participants answered at least “important” to the question if religion was important in coping with their illness (15.4%: of no importance, 15.7%: of some importance, 21.5%: important, 20.6%: very important, 26.7% essential).

### Association Between Study Variables

Female gender was negatively correlated with depression symptoms, *r*_*pb*_ (362) = − 0.13, *p* < 0.05. Malay ethnicity was positively correlated with all three distress measures of depression symptoms, *r*_*pb*_ (362) = 0.11, *p* < 0.05, anxiety symptoms, *r*_*pb*_ (362) = 0.15, *p* < 0.01, and stress symptoms, *r*_*pb*_ (361) = 0.11, *p* < 0.05. Being married or in a relationship was positively correlated with anxiety symptoms, *r*_*pb*_ (362) = 0.11, *p* < 0.05, and stress symptoms, *r*_*pb*_ (361) = 0.12, *p* < 0.05. Having secondary education was positively correlated with all three distress measures of depression symptoms, *r*_*pb*_ (362) = 0.11, *p* < 0.05, anxiety symptoms, *r*_*pb*_ (362) = 0.17, *p* < 0.01, and stress symptoms, *r*_*pb*_ (361) = 0.11, *p* < 0.05. Having a religious affiliation to Islam was also positively correlated with all three distress measures of depression symptoms, *r*_*pb*_ (357) = 0.13, *p* < 0.05, anxiety symptoms, *r*_*pb*_ (357) = 0.16, *p* < 0.01, and stress symptoms, *r*_*pb*_ (356) = 0.13, *p* < 0.05. Lastly, having a religious affiliation to Hinduism was negatively correlated with depression symptoms, *r*_*pb*_ (357) = − 0.11, *p* < 0.05, and anxiety symptoms, *r*_*pb*_ (357) =− 0.14, *p* < 0.01. (Table 2).

**Table 2:** Pearson product moment, point-biserial and phi coefficient statistics between study variables

Coefficients																				
	1	2	3	4	5	6	7	8	9	10	11	12	13	14	15	16	17	18	19	20
1	1.0	0.0	0.0	0.0	− 0.1*	0.1	0.0	0.2**	0.1	0.0	0.0	− 0.1	0.1	0.0	− 0.1	0.0	0.1	0.1	0.0	0.1
2	0.0	1.0	− 0.2**	− 0.2**	0.0	0.0	0.0	0.1	0.1	− 0.1	0.1	0.0	0.0	0.1	0.0	0.0	0.0	− 0.2**	0.1	0.0
3	0.0	− 0.2**	1.0	− 0.1*	0.0	0.0	0.0	0.2**	0.1	0.0	0.1*	− 0.1	0.0	0.0	0.0	0.0	0.1	− 0.1	0.3**	0.1*
4	0.0	− 0.2**	− 0.1*	1.0	0.0	0.1	0.0	0.1	0.1	0.1**	0.1*	− 0.1*	0.1*	0.0	− 0.1	0.0	0.1*	0.0	0.5**	0.1
5	− 0.1*	0.0	0.0	0.0	1.0	− 0.2**	− 0.1	0.0	0.1	0.1	0.2**	0.0	− 0.3**	− 0.3**	0.9**	− 0.1	0.0	0.0	0.0	0.1**
6	0.1	0.0	0.0	0.1	− 0.2**	1.0	− 0.1	0.0	0.0	0.0	− 0.1	0.0	− 0.1*	− 0.2**	0.0	0.6**	0.2**	0.0	− 0.1	0.0
7	0.0	0.0	0.0	0.0	-0.1	-0.1	1.0	0.0	0.1	0.0	0.1	− 0.1	0.1	− 0.1	0.1*	0.0	0.0	− 0.1	0.0	0.1*
8	0.2**	0.1	0.2**	0.1	0.0	0.0	0.0	1.0	− 0.1*	0.1	− 0.1	− 0.1	0.0	0.0	0.0	0.0	0.1	0.1	0.0	0.0
9	0.1	0.1	0.1	0.1	0.1	0.0	0.1	− 0.1*	1.0	0.0	0.1	− 0.1	0.0	0.0	0.1	0.0	0.0	0.0	0.1	0.0
10	0.0	− 0.1	0.0	0.1**	0.1	0.0	0.0	0.1	0.0	1.0	− 0.1	− 0.1**	0.0	0.0	0.1	0.0	0.0	0.0	0.2**	0.0
11	0.0	0.1	0.1*	0.1*	0.2**	− 0.1	0.1	− 0.1	0.1	− 0.1	1.0	− 0.5**	− 0.1	0.0	0.2**	− 0.1	0.0	− 0.1	0.2**	0.1
12	− 0.1	0.0	− 0.1	− 0.1*	0.0	0.0	− 0.1	− 0.1	− 0.1	− 0.1**	− 0.5**	1.0	0.0	0.1	0.0	0.0	0.1	0.0	− 0.1	0.0
13	0.1	0.0	0.0	0.1*	− 0.3**	− 0.1*	0.1	0.0	0.0	0.0	-0.1	0.0	1.0	− 0.4**	− 0.4**	− 0.2**	− 0.1	0.0	0.2**	0.2**
14	0.0	0.1	0.0	0.0	− 0.3**	− 0.2**	− 0.1	0.0	0.0	0.0	0.0	0.1	− 0.4**	1.0	− 0.3**	− 0.1*	0.0	0.0	− 0.1	− 0.1*
15	− 0.1	0.0	0.0	− 0.1	0.9**	0.0	.1*	0.0	0.1	0.1	0.2**	0.0	− 0.4**	− 0.3**	1.0	− 0.1*	0.0	− 0.1	0.0	0.2**
16	0.0	0.0	0.0	0.0	− 0.1	0.6**	0.0	0.0	0.0	0.0	− 0.1	0.0	− 0.2**	− 0.1*	− 0.1*	1.0	0.0	0.0	0.0	0.1
17	0.1	0.0	0.1	0.1*	0.0	0.2**	0.0	0.1	0.0	0.0	0.0	0.1	-0.1	0.0	0.0	0.0	1.0	0.1*	0.1	0.0
18	0.1	− 0.2**	− 0.1	0.0	0.0	0.0	− 0.1	0.1	0.0	0.0	− 0.1	0.0	0.0	0.0	− 0.1	0.0	0.1*	1.0	− 0.1**	0.0
19	0.0	0.1	0.3**	0.5**	0.0	− 0.1	0.0	0.0	0.1	0.2**	0.2**	− 0.1	0.2**	− 0.1	0.0	0.0	0.1	− 0.1**	1.0	0.2**
20	0.1	0.0	0.1*	0.1	0.2**	0.0	0.1*	0.0	0.0	0.0	0.1	0.0	0.2**	− 0.1*	0.2**	0.1	0.0	0.0	0.2**	1.0
21	0.1*	0.1	0.0	0.0	0.2**	− 0.1	0.0	− 0.1	0.0	0.1	0.1*	0.0	− 0.1	0.1	0.1**	− 0.1	0.0	0.0	0.0	0.0
22	0.0	0.1	0.1	0.1	0.0	0.0	0.0	0.0	0.0	0.1	0.0	0.0	0.4**	− 0.2**	0.1	0.0	0.0	0.1	0.2**	0.3**
23	0.0	0.0	0.0	0.2**	0.1*	0.1	0.1	0.1	0.0	0.0	0.0	− 0.1	0.2**	− 0.2**	0.2**	0.0	0.0	0.1	0.1**	0.4**
24	0.0	0.0	0.1*	0.1*	0.1*	0.1	0.0	0.1**	0.1	− 0.1	0.1	− 0.1	0.2**	− 0.2**	0.2**	0.0	0.0	0.0	0.2**	0.5**
25	0.0	0.0	0.1*	0.1*	0.3**	0.0	0.0	0.1	0.1	0.1	0.1**	− 0.1	0.2**	− 0.3**	0.4**	0.0	0.0	0.0	0.2**	0.5**
26	− 0.2**	0.0	0.0	0.0	0.1*	0.0	0.1	0.1	0.0	0.1	0.1	0.0	0.1	− 0.1*	0.2**	− 0.1	− 0.1	0.0	0.1*	0.1*
27	− 0.1*	− 0.1	0.0	0.0	0.1*	0.0	0.0	0.1	0.0	0.0	0.1*	− 0.1	− 0.1	0.0	0.1*	− 0.1*	0.0	0.1	0.0	− 0.1
28	− 0.1	− 0.1	0.0	0.0	0.1**	− 0.1	0.0	0.1*	0.0	0.0	0.2**	0.0	0.0	− 0.1	0.2**	− 0.1**	0.0	0.1	0.1	0.0
29	− 0.1	− 0.1	0.0	0.0	0.1*	0.0	0.0	0.1*	0.0	0.0	0.1*	0.0	0.0	− 0.1	0.1*	− 0.1	0.0	0.1	0.0	0.0

Coefficients									
	21	22	23	24	25	26	27	28	29
1	0.1*	0.0	0.0	0.0	0.0	− 0.2**	− 0.1*	− 0.1	− 0.1
2	0.1	0.1	0.0	0.0	0.0	0.0	− 0.1	− 0.1	− 0.1
3	0.0	0.1	0.0	0.1*	0.1*	0.0	0.0	0.0	0.0
4	0.0	0.1	0.2**	0.1*	0.1*	0.0	0.0	0.0	0.0
5	0.1**	0.0	0.1*	0.1*	0.3**	0.1*	0.1*	0.1**	0.1*
6	− 0.1	0.0	0.1	0.1	0.0	0.0	0.0	− 0.1	0.0
7	0.0	0.0	0.1	0.0	0.0	0.1	0.0	0.0	0.0
8	− 0.1	0.0	0.1	0.1**	0.1	0.1	0.1	0.1*	0.1*
9	0.0	0.0	0.0	0.1	0.1	0.0	0.0	0.0	0.0
10	0.1	0.1	0.0	-0.1	0.1	0.1	0.0	0.0	0.0
11	0.1*	0.0	0.0	0.1	0.1**	0.1	0.1*	0.2**	0.1*
12	0.0	0.0	− 0.1	− 0.1	− 0.1	0.0	− 0.1	0.0	0.0
13	− 0.1	0.4**	0.2**	0.2**	0.2**	0.1	− 0.1	0.0	0.0
14	0.1	− 0.2**	− 0.2**	− 0.2**	− 0.3**	− 0.1*	0.0	− 0.1	− 0.1
15	0.1**	0.1	0.2**	0.2**	0.4**	0.2**	0.1*	0.2**	0.1*
16	− 0.1	0.0	0.0	0.0	0.0	− 0.1	− 0.1*	− 0.1**	− 0.1
17	0.0	0.0	0.0	0.0	0.0	− 0.1	0.0	0.0	0.0
18	0.0	0.1	0.1	0.0	0.0	0.0	0.1	0.1	0.1
19	0.0	0.2**	0.1**	0.2**	0.2**	0.1*	0.0	0.1	0.0
20	0.0	0.3**	0.4**	0.5**	0.5**	0.1*	− 0.1	0.0	0.0
21	1.0	0.0	− 0.1	0.0	0.1	0.1	0.1	0.0	0.0
22	0.0	1.0	0.5**	0.4**	0.5**	0.2**	− 0.1	0.0	0.0
23	− 0.1	0.5**	1.0	0.5**	0.5**	0.2**	0.0	0.1	0.0
24	0.0	0.4**	0.5**	1.0	0.7**	0.3**	0.1	0.2**	0.2**
25	0.1	0.5**	0.5**	0.7**	1.0	0.5**	0.1*	0.3**	0.2**
26	0.1	0.2**	0.2**	0.3**	0.5**	1.0	0.3**	0.3**	0.4**
27	0.1	− 0.1	0.0	0.1	0.1*	0.3**	1.0	0.7**	0.8**
28	0.0	0.0	0.1	0.2**	0.3**	0.3**	0.7**	1.0	0.8**
29	0.0	0.0	0.0	0.2**	0.2**	0.4**	0.8**	0.8**	1.0

* Indicates significance at *p* < 0.05. ** indicates significance at *p* < 0.01

1: Gender Female; 2: Age group 35 – 44; 3: Age group 45 – 54; 4: Age group 55 – 65; 5: Ethnicity Malay; 6: Ethnicity Indian; 7: Ethnicity Others; 8: Relationship status Married / In a relationship; 9: Relationship status Divorced / Separated / Widowed; 10: Highest education level Primary and below; 11: Highest education level Secondary; 12: Highest education level Pre-tertiary; 13: Religious affiliation Christianity; 14: Religious affiliation Buddhism / Taoism; 15: Religious affiliation Islam; 16: Religious affiliation Hinduism; 17: Religious affiliation Sikhism; 18: Primary diagnosis Affective psychosis; 19: Duration of illness (years); 20: Religion important in coping with illness; 21: Belief that religion is incompatible with psychiatric treatment; 22: ORA score; 23: NORA score; 24: IR score; 25: PRC; 26: NRC; 27: DASS21 Depression symptoms; 28: DASS21 Anxiety symptoms; 29: DASS21 Stress symptoms

### Association Between DUREL, Religious Coping Patterns, and Patient Distress

Three separate linear regressions were run, investigating the relationship between ORA, NORA, IR, religious coping (positive and negative), and the three aspects of distress (depression symptoms, anxiety symptoms, and stress symptoms) as the outcome variables. Gender, marital status, highest education level, and religious affiliation were adjusted for in these analyses (Table 3). The ethnicity of the participants was not included as a predictor variable as it had a high variable inflation factor (VIF) of above 10, together with religious affiliation in the model, indicating multicollinearity. This was likely because all the participants who identified themselves as Malay (*n* = 57) also identified Islam as their religious affiliation.

**Table 3:** Multiple linear regression models of the relationship between religious coping, and distress (depression, anxiety and stress symptoms on the DASS-21 scale)

Variables	Depression symptoms				Anxiety symptoms				Stress symptoms			
	Β	SE	*t*	*p*	β	SE	*t*	*p*	β	SE	*t*	*p*
Organised Religious Activity (ORA)	− 0.82	0.47	− 1.76	0.080	− 0.99	0.40	− 2.49	**0.013**	− 0.83	0.45	− 1.83	0.068
Non-organised Religious Activity (NORA)	− 0.18	0.38	− 0.46	0.643	− 0.01	0.33	− 0.03	0.975	− 0.17	0.37	− 0.46	0.646
Intrinsic Religiosity (IR)	0.30	0.23	1.31	0.192	0.45	0.20	2.28	0.023	0.45	0.23	1.97	0.050
Positive Religious Coping (PRC)	− 0.05	0.13	− 0.40	0.688	0.15	0.11	1.31	0.191	0.08	0.13	0.59	0.559
Negative Religious Coping (NRC)	0.66	0.12	5.67	** < 0.001**	0.38	0.10	3.86	** < 0.001**	0.61	0.11	5.38	** < 0.001**

Regression models are adjusted for gender, marital status, highest education level, and religious affiliation

Bold print highlights statistically significant *p* values at *p* < 0.017

ORA was negatively and significantly associated with the outcome variable of anxiety symptoms (*β* = − 0.99, *p* = 0.013). For all the three models, negative religious coping was positively and significantly associated with the outcome variables of depression symptoms (*β* = 0.66, *p* < 0.001), anxiety symptoms (*β* = 0.38, *p* < 0.001) and stress symptoms (*β* = 0.61, *p* < 0.001). Positive religious coping, however, was not significantly associated with any of the three outcomes in the models.

## Discussion

With the majority of the sample reporting a belief in God or a Higher Power (78.6%) and religion being important in coping with their illness (68.9%), religion seems to play an important role in the lives of the majority of the outpatients with psychosis in Singapore, supporting the study hypothesis. These results are consistent with a larger study conducted on 1800 respondents in Singapore in the general population that found the majority of the population to have a belief in a God or higher power (Mathews et al., 2019) and a Gallup poll that indicated that about 70% of Singaporeans adults indicated that religion was important in their daily lives (Crabtree, 2010). In comparison with a western population, the percentage of respondents who stated that religion was important in coping with their illness in the current sample (68.9%) was higher than that of a sample of outpatients with schizophrenia in Geneva, Switzerland (58%) (Huguelet et al., 2006). The mean scores of PRC (18.5 ± 6.9) and NRC (12.3 ± 5.3) reported in the study are also higher than that reported in a sample of outpatients with schizophrenia in the United States for both PRC (15.6 ± 4.18) and NRC (5.21 ± 3.76) (Nolan et al., 2012). It was also higher than that reported in a sample of outpatients with schizophrenia in India for both PRC (14.6) and NRC (8.3) (Triveni et al., 2017). While there seems to be a general trend of religion being more important in the daily lives of people living in low-income countries, countries such as the United States, Singapore, Italy, and Greece appear to go against this trend (Crabtree, 2010). Hence, it may not be appropriate to reduce the importance of religion to patients based on geography or income level alone, as other socio-political reasons may contribute to the amount of value a society attributes to religion. Nonetheless, overall, the current study results reflect the importance of religion to outpatients with psychosis in Singapore, while the high mean scores on NRC point to potential areas of concern for clinicians to address, especially given the association between NRC and greater distress scores.

The findings of negative religious coping and its association with all three symptoms of distress (depression, anxiety, and stress) support the study hypothesis. These findings were also reported in a similar study conducted in Malaysia (Nurasikin et al., 2013) and a study conducted in the United States (Rosmarin et al., 2013). The findings suggest that employing negative religious coping methods might significantly impact distress among patients with psychosis. Negative religious coping is characterised by “signs of spiritual tension, conflict and struggle with God and others, as manifested by negative reappraisals of God’s powers (e.g., feeling abandoned or punished by God), demonic reappraisals (*i.e.*, feeling the devil is involved in the stressor), spiritual questioning and doubting, and interpersonal religious discontent” (Pargament et al., 2011). As such, it is not difficult to see how such negative representations of God / a higher power might lead to hopelessness and despair that might be reflected in the greater distress symptoms reported. While not measured in the current study, NRC has been associated with greater suicidal ideations (Rosmarin et al., 2013), further emphasising NRC’s damaging emotional and psychological effects. In sum, the current results support the need to address NRC methods in therapy to alleviate patient distress.

Contrarily, against the study hypothesis, there were no significant associations found between positive religious coping and symptoms of distress. In their review of 29 studies that used the Brief RCOPE to measure PRC and NRC methods, Pargament and colleagues (2011) found that PRC was consistently associated with greater well-being but not consistently with negative constructs. This is coherent with the current study that did not find any significant relationship between PRC and the negative constructs of greater distress symptoms. PRC might hence play a bigger role in improving overall well-being (not measured in the current study) than alleviating negative outcomes such as distress symptoms. Nonetheless, the interpretation of the significant associations in the present study must be made cautiously due to the cross-sectional nature of this study. It might also be possible that patients facing greater distress (depression, anxiety and stress symptoms) due to their psychopathology or living conditions are more likely to view God or a Higher Power in a negative light (for example: abandoning them or punishing them for something they did) as an attribution for their negative circumstances.

Our finding that religious attendance was associated with lower anxiety symptoms is consistent with the extant literature on non-psychiatric populations (Ellison et al., 2009; Jansen et al., 2010; Sternthal et al., 2010). Thus, it might be possible that ORA protects against anxiety due to the social and emotional support offered by the religious community. Aliche and colleagues (2020), in their study, suggest that religious attendance might help in emotion regulation (by way of reconstruction of the meaning of one’s illness) which might reduce anxiety symptoms. However, due to the cross-sectional design of the current study, temporal relationships cannot be affirmed as it might also be possible that increased anxiety symptoms might promote social withdrawal and hence impede participation in ORA.

The importance of religion in coping with their illness reported in this study and the association reported between NRC and depression, anxiety, and stress symptoms, adds weight to recent studies and suggests the need for clinicians to address the religious beliefs of their clients in practice (Koenig, 2008). This is supported by previous research that also suggests the importance of religion in coping and patients’ desire for their religious needs to be considered in clinical care (D’Souza, 2016). Mizock and colleagues (2012) have delineated steps that psychotherapists might take to integrating religious perspectives in psychotherapy and provide religiously sensitive care. These steps include training in the domain of the clients’ religious belief systems and rituals, inquiring about and assessing the role of the religion in the lives of their clients, and lastly, implementation. Implementation includes steps such as “formulation and understanding of individual’s (religious) goals and making connections to appropriate resources in therapy.” However, it must be noted that some clinical establishments might not have the adequate resources to train psychotherapists in the domain of the religious belief systems of the clients, or some clients might have unique religious needs that warrant greater expertise to address them. Clients in such scenarios could alternatively be referred to “religious organisations or groups, clergy, religious leaders or other facilitators of religious processes, exercising caution to ensure that the referral is likely to be helpful and not harmful” (Mizock et al., 2012). These groups or individuals could work together with the clinical teams, considering clients’ spiritual concerns and needs, thus treating clients more holistically.

The main strength of the current study is that it is one of the few in Asia and multi-ethnic populations. To the best of our knowledge, it is the first in Singapore to investigate the role religion plays in the population with psychosis. It provides valuable information for clinical practice, emphasising the importance of religion among patients with psychosis. Additionally, with the lack of research on how religiosity (ORA, NORA, and IR) might influence distress symptoms amongst those with psychosis, the current study provides early findings worth further investigation. While the current research has its strengths, it is not without its limitations. In the current research, due to the lack of available validated instruments in non-English languages, participants who were not literate in English were excluded from the study. The results of the current study might hence not be generalisable to these populations. Additionally, participants of each religious affiliation group were deemed a homogeneous sample, whereas in reality, there might be differences in how one perceives their relationship with God or a higher power based on their specific religious community (within the major religious groups).

## Conclusion

This study demonstrates that religion plays an important role in both coping and the psychopathology of outpatients seeking help for psychosis in a tertiary care setting in Singapore. While increasingly research has focused on the relationship between religion and outcomes (both positive and negative) through the pattern of religious coping used, little research has investigated why certain patients are more likely to employ a specific type of religious coping pattern. This information might be helpful when addressing the underlying causes for using negative coping methods in psychotherapeutic practice. Nonetheless, the current findings reinforce the burgeoning voice to integrate religiosity in mental health care.

## Data Availability

The datasets and material used and/or analysed during the current study are available from the corresponding author on reasonable request.

## Footnotes

1 REDCap (Research Electronic Data Capture) is a secure, web-based software platform designed to support data capture for research studies, providing (a) an intuitive interface for validated data capture; (b) audit trails for tracking data manipulation and export procedures; (c) automated export procedures for seamless data downloads to common statistical packages; and (d) procedures for data integration and interoperability with external sources.
